# Pattern-tunable synthetic gauge fields in topological photonic graphene

**DOI:** 10.1515/nanoph-2021-0647

**Published:** 2022-03-10

**Authors:** Zhen-Ting Huang, Kuo-Bin Hong, Ray-Kuang Lee, Laura Pilozzi, Claudio Conti, Jhih-Sheng Wu, Tien-Chang Lu

**Affiliations:** Department of Photonics and Institute of Electro-Optical Engineering, College of Electrical and Computer Engineering, National Yang Ming Chiao Tung University, Hsinchu 30050, Taiwan, ROC; Institute of Photonics Technologies, National Tsing Hua University, Hsinchu 30013, Taiwan, ROC; Physics Division, National Center for Theoretical Sciences, Hsinchu 30013, Taiwan, ROC; Institute for Complex Systems, National Research Council (ISC-CNR), Via dei Taurini 19, 00185 Rome, Italy; Research Center Enrico Fermi, Via Panisperna 89a, 00184 Rome, Italy; Department of Physics, University Sapienza of Rome, Piazzale Aldo Moro 5, Rome 00185, Italy

**Keywords:** chiral strain-engineering, strong localization, synthetic gauge fields, topological edge state, tunable capability

## Abstract

We propose a straightforward and effective approach to design, by pattern-tunable strain-engineering, photonic topological insulators supporting high quality factors edge states. Chiral strain-engineering creates opposite synthetic gauge fields in two domains resulting in Landau levels with the same energy spacing but different topological numbers. The boundary of the two topological domains hosts robust time-reversal and spin-momentum-locked edge states, exhibiting high quality factors due to continuous strain modulation. By shaping the synthetic gauge field, we obtain remarkable field confinement and tunability, with the strain strongly affecting the degree of localization of the edge states. Notably, the two-domain design stabilizes the strain-induced topological edge state. The large potential bandwidth of the strain-engineering and the opportunity to induce the mechanical stress at the fabrication stage enables large scalability for many potential applications in photonics, such as tunable microcavities, new lasers, and information processing devices, including the quantum regime.

## 1 Introduction

Topologically protected edge states with the immunity to distortions or fabrication imperfections of crystals have attracted much attention in recent years. Since the first discovery of the quantum Hall effect was reported [1], the conductive electronic state located at the structural boundary has become a popular topic in many electronic applications [2], [3], [4], [5]. However, to induce the quantum Hall effect, a strong magnetic field is necessary. The field induces cyclotron motion and forms quantized electronic states called Landau levels in the bulk region. As a result, an edge state with broken time-reversal symmetry is presented in the system. In 2005, a breakthrough was discovered and proposed by Kane and Mele [6], who outlined that spin-dependent topological states without involving any external magnetic field may occur between K and K′ point of the Brillouin zone in graphene. This notable feature pushes the topological edge state into practical applications as the pivotal building block for the development of topological insulators. Based on the superior transport properties of the massless Dirac fermions and topological protection, topological insulators have exhibited outstanding performance in topological superconductors [2, 7, 8], spintronics devices [3], and quantum computing [5]. In comparison to electrons, the coupling between the magnetic field and photons is weaker. Therefore, the spin-orbital coupling is usually used to create band inversion and change the photonic system’s topology [9], [10], [11]. The corresponding topological edge state possesses the time-reversal symmetry and is protected from backscattering by spin-momentum locking [9, 12, 13], with inhibition of experimental fluctuations. Thanks to the unprecedented features, the photonic topological insulator has exhibited extraordinary performance in many optical devices, including unidirectional waveguides [14, 15], optical switching [16, 17], optical isolators [18], and lasers [19], [20], [21].

Mainly, most designs of topological photonics either utilized magnetic materials or phase resonators, which usually operate at low frequencies and lack scalability. Interestingly, based on spin-orbital coupling, a synthetic gauge theory was proposed to mimic the quantum Hall effects in the absence of an external magnetic field [22, 23]. The synthetic gauge field is created by the appearance of a continuously distributed strain, whose curl gives an artificial magnetic field. Notably, in the presence of the strain-induced pseudo-magnetic field, a photonic bandgap can be opened, and Landau levels appear in the band diagram of the photonic system [24], [25], [26]. Note that strain in these photonic systems is not due to external force but is used to describe designed patterns by fabrication. We call it pattern-tunable strain. These photonic Landau levels have a large group index and high density of state (DOS), leading to a strong Purcell effect and increasing the light–matter interaction. Additionally, the frequency gap between two Landau levels provides the insulating capability in the bulk region. According to the bulk-edge correspondence in the quantum Hall effect, there should be an edge state inside this frequency gap. However, only a few studies have successfully demonstrated the topological edge state under the pseudo-magnetic field [27]. Compared to other edge states constructed by breaking the parity symmetry [21, 28], the strain-induced edge state with an off-Γ momentum shows a shorter propagation length due to significant diffraction loss near the structural boundary, which drastically limits its applicability [24, 27]. Another limitation of edge states of a strained system at the interface between vacuum and the lattice is that these edge states are not robust against various lattice terminations. This is because some types of lattice terminations will cause intervalley couplings and break the topologies. To make the strain-induced topological edge state stable enough for practical application, it is mandatory to have a design that reduces diffraction loss and avoids intervalley couplings. For example, an armchair boundary will mix two valleys. This motivates us to consider a two-domain system of smooth strain modifications.

**Figure 1:**
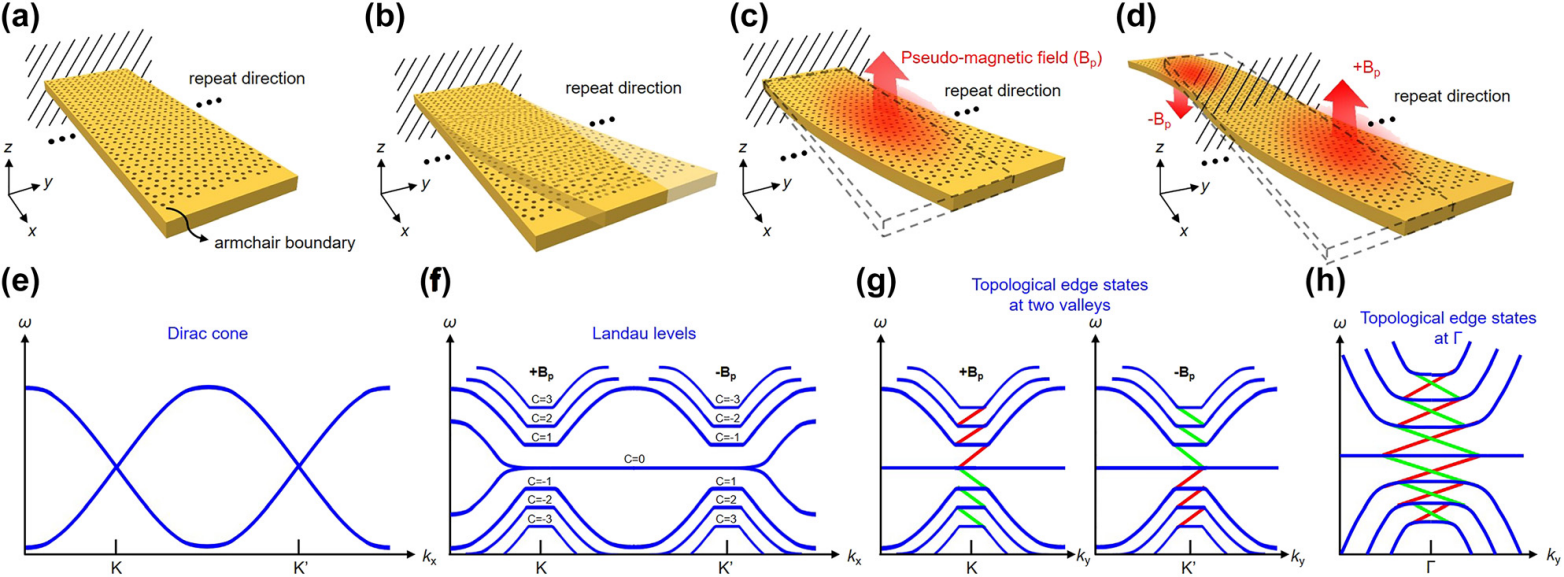
The schematic diagrams of deformation processes of a membrane containing honeycomb superlattice. (a) Before applying the strain constituted by shifting the lattice arrangement, a membrane with a honeycomb lattice is fixed like a cantilever beam. (b) After applying the strain, the membrane is deformed along the *y*-axis with one end fixed. (c) A synthetic vector potential is produced by the continuously distributed strain, and its curl can define a pseudo-magnetic field *B*_p_. (d) A chiral structure, which is composed of two patterns with opposite pseudo-magnetic fields. (e) The band diagram of the membrane before applying the strain, and two Dirac cones exist at the K and K′ valley. (f) After applying the strain, bands split into several Landau levels, and a local bandgap appears in the band diagram. Also, the corresponding valley Chern numbers (*C*) are indicated in this figure, and the central flat band represents the 0th photonic Landau level. (g) The band diagram of the chiral structure. After the combination of two patterns with opposite pseudo-magnetic fields, topological edge states appear between each Landau level at the K and K′ valley, where the red and green solid lines are indicated as the spin-up and spin-down edge states, respectively, and the photon spin is defined by the direction of the circular polarization. (h) Topological edge states at the K and K′ valley are simultaneously projected to the Γ point based on Bloch’s band theory because the direction of lattice arrangement along the *x*-axis of the membrane is along the zigzag.

Here, we propose a chiral structure obtained by a continuous displacement function in the arrangement of holes in a honeycomb superlattice. The displacement function corresponds to deformations sketched in Figure 1, where the structure is periodic along the *y*-direction and finite along the *x*-direction. Although the structure is finite in the *x*-direction, the number of unit cells in the *x*-direction is large enough to maintain the physics of the Dirac cones in the K and K′ valleys. Figure 1(a) shows a membrane with air holes arranged in a honeycomb lattice fixed at one end like a cantilever beam. Note that strain in this work does not mean the relative displacement due to external force but refers to the intentional arrangements of the positions of the air holes, as shown in Figure 1(b). In Figure 1(c), the strain of air holes in the membrane produces a synthetic vector potential, whose curl determines the strength and direction of the pseudo-magnetic field. Even though strain breaks the C3 symmetry of the lattice, the effects of strain can be replaced by a pseudo-magnetic field. Thus, the strained system behaves as an unstrained system in the pseudo-magnetic field [29]. Remarkably, when increasing the strength, a local bandgap is opened and gradually broadened at the first Dirac cone of the honeycomb lattice (Figure 1(e)), and Landau levels with different valley Chern numbers (*C*) form at each K and K′ valley in the band diagram (Figure 1(f)) [30], as in the quantum Hall effect but still preserving the time-reversal symmetry. Accordingly, the strain-induced pseudo-magnetic field must be reversed between K and K′ valley to maintain the time-reversal symmetry in the global Dirac Hamiltonian [31]. Figure 1(d) shows a chiral structure composed of two strained patterns with the pseudo-magnetic field in opposite directions. Owing to the reverse topology resulting from the opposite pseudo-magnetic fields, topological edge states appear between different orders of Landau levels (Figure 1(g)), where the red and green solid line represents the spin-up and spin-down edge state, respectively. These modes are spatially located at the boundary between the two domains. Furthermore, if the *x*-direction shown in Figure 1(d) is along the zigzag, the bands at K and K′ valley after the deformation would be simultaneously projected to the Γ point based on Bloch’s band theory. Subsequently, the crossing bands, which include the spin-up edge state in one valley and spin-down edge state in the other valley, appear between Landau levels and belong to the radiation mode because these bands are in the light cone, as shown in Figure 1(h). It is worth noting that tuning the magnitude and peak position of the pseudo-magnetic field, a highly localized strain-induced topological edge state at Γ point can be obtained.

The mode localization of strain-induced topological edge states is significantly enhanced because of the continuous modulation, which implies the diffraction loss of the strain-induced topological edge states can be extremely reduced with respect to the edge state of the 0th Landau level [27]. This method provides an effective way to design lossless edge states which also exhibits scattering-free characteristics. Additionally, strain-engineering applies to all the frequencies and is suitable for photonics since strains can be arranged on purpose at the fabrication stage. With such performance, our results pave the way for the practical application of strain-induced topological edge states and are beneficial for low-energy-consumption optical devices.

## 2 Theories and methods

**Figure 2:**
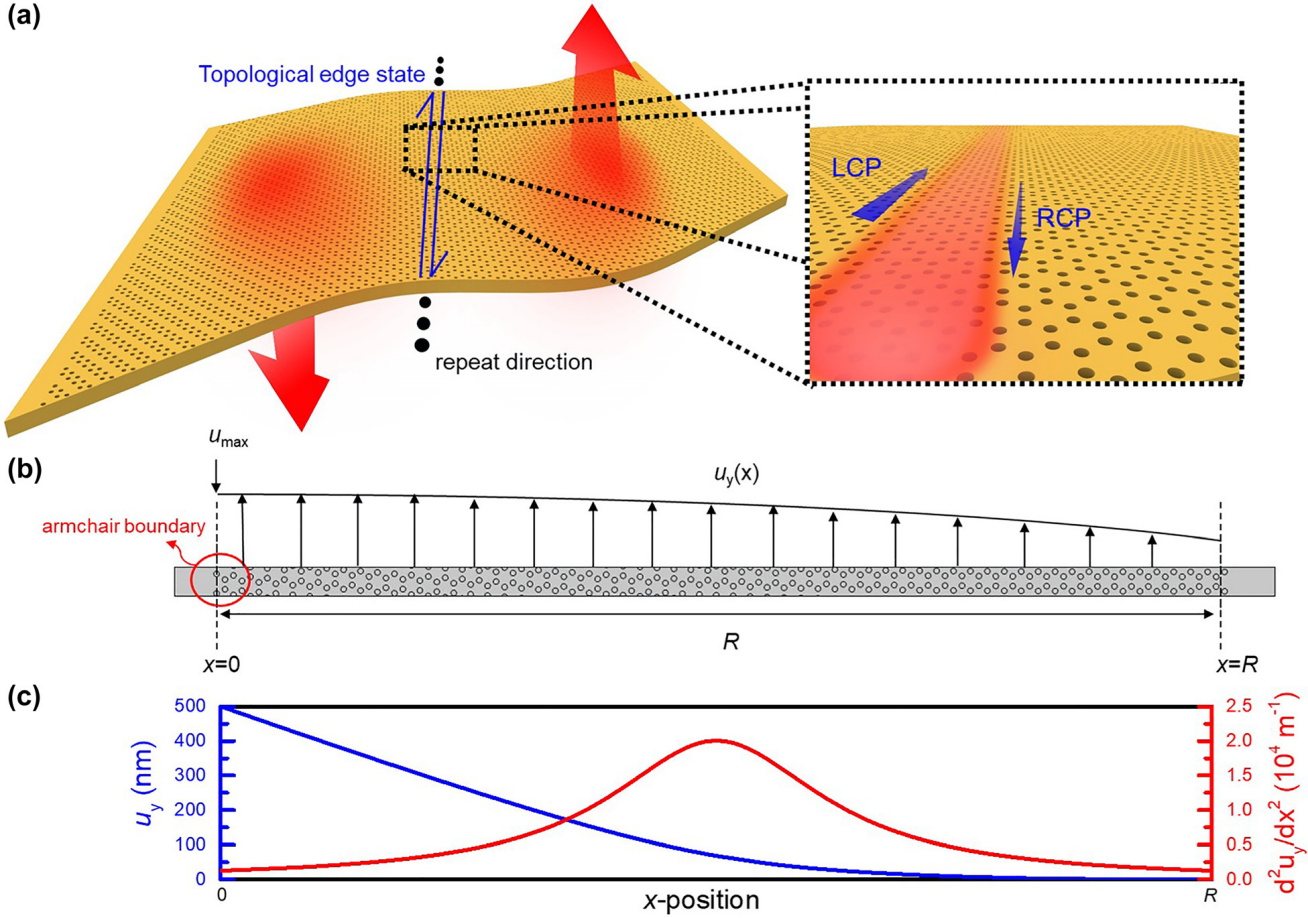
The schematic diagrams of the proposed chiral structure and the applying displacement. (a) The schematic diagram of the chiral structure, which supports the strain-induced topological edge state. (b) The schematic diagram of the honeycomb superlattice and the continuous *y*-displacement, which is the function of *x* and distributes from *x* = 0 to *x* = *R*, and the *x*-axis is along the zigzag direction of the honeycomb lattice. (c) The continuous displacement functions. The blue line indicates the distribution of *u*_y_, and the red line indicates the distribution of *∂* ^2^ *u*_*y*_/∂*x* ^2^.

The unstrained system is made of a GaAs membrane with air holes disposed in a honeycomb lattice with an armchair boundary. The operation energy we focused on here is near 1.32 eV, which corresponds to the photoluminescence band of GaAs, and the refractive index of GaAs is set to 3.5. As shown in Figure 2(a), the strained system is obtained by adding a continuous displacement function to the arrangement of air holes. Given the strain (*ε*)-displacement (*u*) relation:

(1) $$\left(\begin{array}{ll} \epsilon_{xx} & \epsilon_{xy}\\ \epsilon_{yx} & \epsilon_{yy} \end{array}\right)=\frac{1}{2}\left(\begin{array}{ll} 2u_{x,x} & u_{x,y}+u_{y,x}\\ u_{y,x}+u_{x,y} & 2u_{y,y} \end{array}\right)$$

the resulting strain distribution for a given displacement function can be obtained. Then, according to the work published by Guinea in 2009 [32], the strain can generate a gauge field A and lead to the Landau quantization at K valley [29, 33], [34], [35], [36]. The effective Hamiltonian near one of the valleys can be described by the following equation:

(2) $$H=\hbar v_{\text{D}}\left(\vec{k}-\vec{A}\right)\cdot \vec{\sigma }$$

where *v*_D_ and *σ* are the group velocity at the Dirac point and Pauli matrices, respectively. Because of time-reversal symmetry, the Hamiltonian of K′ valley is simply the negative of Eq. (2). Furthermore, the relation between the gauge field and strain is shown as follows [32],

(3) $$\left(\begin{array}{l} A_{x}\\ A_{y} \end{array}\right)=g_{2}\left(\begin{array}{l} \epsilon_{xx}-\epsilon_{yy}\\ -2\epsilon_{xy} \end{array}\right)$$

where the *x*-direction is along the zigzag, the *y*-direction is along the armchair, and *g*_2_ is a material-related parameter. Originally, using an effective A to describe the effects of strain was proposed for tight-binding systems. To exam the validity of using Eq. (2) in photonic systems with shifted holes, we calculate the shifts of Dirac points in the reciprocal space due to uniform strain in our systems. The results confirm the shifts of the Dirac points by strain, and hence the plausibility of using Eq. (2) (see the details in the Supplementary Material). In our honeycomb lattice, the $\sqrt{3}a$ is chosen as 350 nm, where *a* is the lattice constant, and the filling factor is chosen as 0.2. To determine the value of *g*_2_, Eq. (3) is considered in the strain-induced gauge field, and then we can substitute it into Figure s6c. The detail calculation is also discussed in Figure s6. By this method, *g*_2_ in our material system can be calculated as 7.07 × 10^6^ m^−1^. Hereafter, the pseudo-magnetic field can be determined by calculating the curl of the synthetic gauge field. To analogize the quantum Hall effect, an out-of-plane magnetic field is necessary. Therefore, we consider only its *z*-component, which implies that *A*_*x*,*y*_ and *A*_*y*,*x*_ will dominate the strain-induced quantum Hall effect. Besides, the edge state in Figure 2(a) is designed to propagate along the *y*-direction, with the lattice preserving the periodicity in the same direction. Thus, we only add an *x*-varied displacement into our system, which means the *A*_*x*,*y*_ term is null. Finally, based on Eqs. (1) and (3), the relation between the displacement and pseudo-magnetic field can be derived as follows:

(4) $$B_{z}=A_{y,x}=-g_{2}u_{y,xx}$$

In Eq. (4), the distribution of the pseudo-magnetic field only depends on the second-order differentiation of *u*_*y*_. If an inflection point is added to the input displacement function, the structure will be divided into two regions. The region with a positive second derivative of the displacement exhibits the pseudo-magnetic field in the +*z*-direction, and the other region exhibits the pseudo-magnetic field in the −*z*-direction. The strain-induced quantum Hall effect in these two regions will display opposite characteristic and then result in a reverse topology. As a consequence, the boundary defined by the inflection point host topological edge states, which are protected from the diffraction loss near the structural boundary, giving a chiral structure with tunable strong localization as designed in Figure 2(a) following the approach by Guinea [32]. However, the strain patterns in [32] seem unlikely to generate propagating edge states. On the contrary, our approach allows high Q edge modes with also tunable localization widths. All the simulations were calculated by using the frequency domain solver of the finite-element software (COMSOL Multiphysics).

## 3 Results

To induce a pseudo-magnetic field, a continuous displacement as a function of the *x*-coordinate in the *y*-direction is applied to the suspended GaAs membrane. The schematic diagram of the displacement function is shown in Figure 2(b). First, we focus on a Lorentzian-distributed pseudo-magnetic field:

(5) $$B_{z}\left(x\right)=-g_{2}\frac{b^{2}}{{\left(x-L\right)}^{2}+b^{2}}$$

where *L* and *b* signify the peak position and the half-width at half-maximum (HWHM), respectively. The Lorentzian-distributed pseudo-magnetic field would give the local response of the strain-induced quantum Hall effect. Then, according to Eq. (4), the displacement function can be inversely derived by substituting some boundary conditions, including fixing the maximum displacement *u*_max_ at *x* = 0, no displacement at *x* = *R*, and the first derivative of the displacement equal 0 at *x* = *R*, where *R* represents the final *x*-coordinate in Figure 2(b). Afterward, a continuous displacement function with a Lorentzian-distributed pseudo-magnetic field can be obtained. Figure 2(c) demonstrates the derived displacement function and its second derivative in the condition of fixing *u*_max_ as 500 nm and *L* as 0.5 × *R*, respectively.

### 3.1 Strain-induced Landau levels

**Figure 3:**
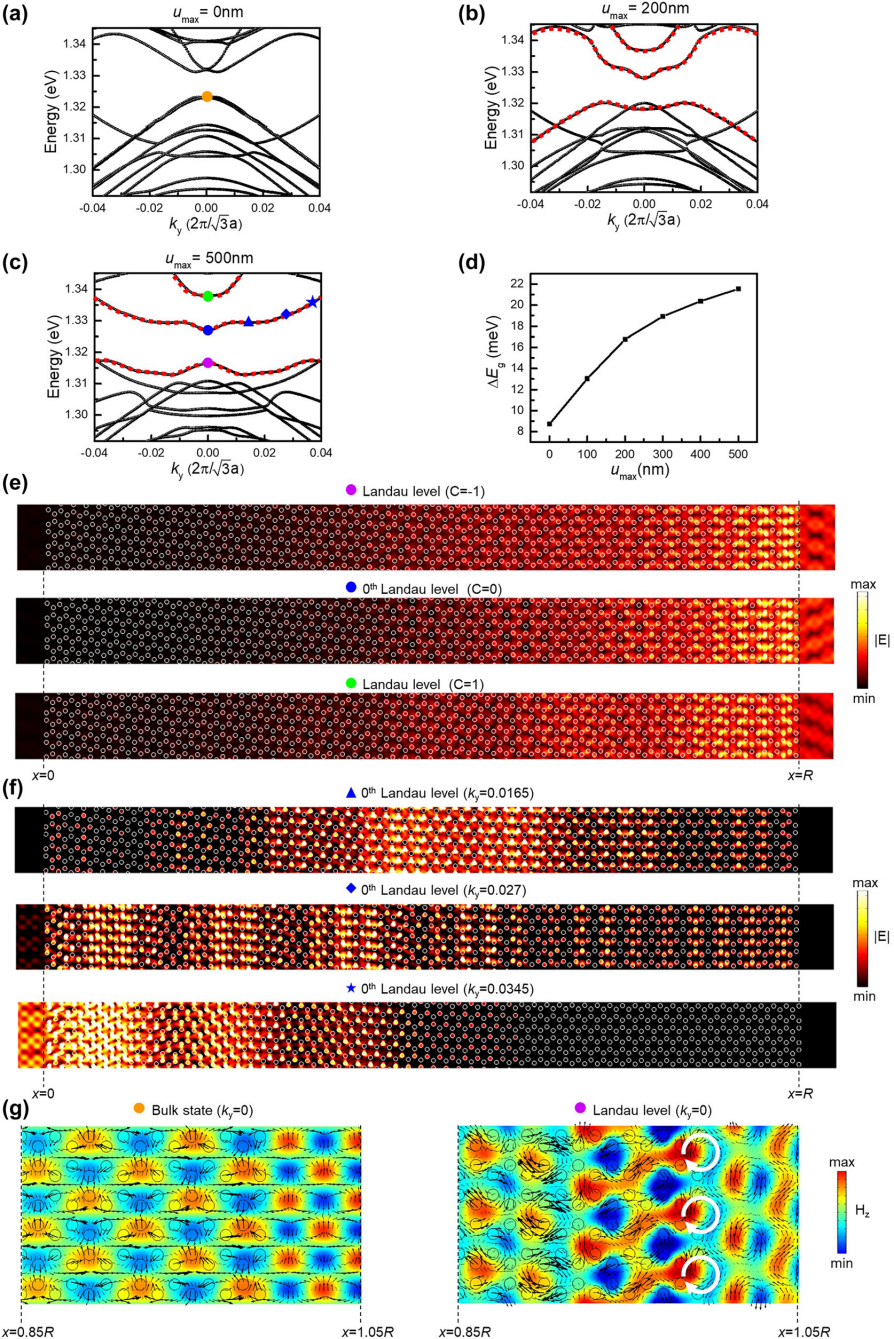
The appearance of photonic Landau levels. (a)–(c) The band diagram when *u*_max_ equals 0 nm (a), 200 nm (b), and 500 nm (c), respectively. The red dot lines in (b) and (c) indicate the photonic Landau levels. (d) Δ*E*_g_ as the function of *u*_max_. (e) The electric field distribution of the three photonic Landau levels at the Γ point and the corresponding valley Chern number. (f) The electric field distributions of the 0th photonic Landau level at 0.0165, 0.027, and 0.0345 of *k*_*y*_ in (c). (g) The distributions of magnetic field in the *z*-component *H*_*z*_ at the right ends of our structures, where the black arrows indicate the in-plane electric field vector. The left side of g is the bulk state of the unstrained structure, and the right side is that of the strained structure, which is also indicated as an orange and purple dot in (a) and (c), respectively.

Based on the derived distribution of the displacement, the band diagram varied with *u*_max_ is shown in Figure 3. The honeycomb supercell in the simulation model is fixed as 60 periods in the *x*-direction, as shown in Figure 2(b). When *u*_max_ is increased in the displacement function, the peak of the second derivative is raised, resulting in an enhancement of the pseudo-magnetic field. Therefore, the strain-induced quantum Hall effect should become more significant as enlarging *u*_max_. Figure 3(a) shows the band diagram in the absence of the input displacement. Although there is no shift in the arrangement of air holes, a small bandgap still appears at the Γ point, which is due to the finite number of periods in the *x*-direction. Additionally, in Figure 3(a), there is no gapless edge state inside the bandgap. For a graphene nanoribbon, gapless edge state always exists along the zigzag boundaries no matter how short the nanoribbon is [37]. Consequently, we change the periodic direction to the zigzag and calculate the band diagram of a short nanoribbon, as shown in Figure s7. In this calculation, even though the width of the nanoribbon is short, the gapless edge state still exists, and its dispersion is nearly not affected by the applying strain. We also realize that there is no robust edge state along the armchair boundaries, and therefore the strain introduced in Figure 1 needs to be applied. As the displacement shown in Figure 2(c) is added, and *u*_max_ is increased to 200 nm, the local bandgap slightly broadens, and three photonic Landau levels indicated as red dot lines with a higher DOS appear in the band diagram, as shown in Figure 3(b). When *u*_max_ continues to be increased to 500 nm, the local bandgap at the Γ point is further expanded, which is as large as 21.5 meV observed in Figure 3(c). Most vitally, increasing *u*_max_ up to 500 nm in the displacement function implies the Lorentzian-distributed pseudo-magnetic field becomes sharper in the spatial distribution, causing a significant local response of the strain-induced quantum Hall effect and an increase in the energy splitting ∆*E*_g_ between photonic Landau levels, as shown in Figure 3(d). This striking phenomenon can be also observed in the variation of DOS of photonic bands in the band diagram. Figure 3(e) shows the electric field distribution of the three photonic Landau levels at the Γ point, and the corresponding valley Chern number is also indicated in each figure. Since our system has a finite width, the calculation of Chern numbers is done by first assuming that the region with a magnetic field can be described by an effective low-energy Hamiltonian near the valley. The detailed discussion of the valley Chern number is demonstrated in the first section of the Supplementary Material. Notably, the mode profile of each photonic Landau level is similar at the Γ point and mainly distributed on the right side of the structure, which is close to the undeformed structural boundary shown in Figure 2(c). According to the nature of Landau levels, the wave function will be changed from the bulk state to the edge state as the *k* along the periodic direction gradually deviates from the reciprocal lattice point [26], which is the Γ point in this work. However, because of the asymmetric deformation we applied, the bulk state at the Γ point also shows the broken geometric symmetry in each photonic Landau level, whose electric field is blocked at the peak position of the pseudo-magnetic field. To verify more detailed features, the electric field distribution of the 0th photonic Landau level at several off-Γ points is further extracted and shown in Figure 3(f). It is obvious to see that when *k*_*y*_ is increased and deviated from the Γ point, the electric field gradually shifts to the left side of the structure, where the strain is primarily distributed. Moreover, a comparison of the polarization between bulk states indicated as the orange and purple dots in Figure 3(a) and (c) is shown in Figure 3(g). The figure only demonstrates the field distribution of the right end of our structure, which matches where the maximum electric field is distributed in Figure 3(e), and the black arrows show the in-plane electric field vector. Surprisingly, when the structure is in the absence of the strain, the in-plane electric fields do not rotate (left side of Figure 3(g)), leading to a zero angular momentum. In contrast, as the strain is applied, the in-plane electric fields start to rotate (right side of Figure 3(g)), resulting in a non-zero angular momentum, which also matches the physics of the quantum Hall effect. Besides, for a Dirac fermion in an external magnetic field *B*_e_, the energy spacing between Landau levels can follow the below equation [38]:

(6) $$\Delta E_{mn}=\hbar v_{D}\sqrt{2B_{e}}\left(\sqrt{m}-\sqrt{n}\right)$$

where *m* and *n* are the order of the Landau level. Based on Eq. (6), the analytical energy spacing between photonic Landau levels can be calculated. Since our numerical calculations are for nonuniform pseudomagnetic fields, the analytical and numerical results do not give close numbers but only the same order of magnitude. According to Eq. (5), the maximum *B*_z_ in Figure 3(b) and (c) are calculated as 3.38 × 10^10^ m^−2^ and 1.42 × 10^11^ m^−2^, respectively, and then we substitute it into Eq. (6) to estimate the energy spacing between the 0th and 1st photonic Landau levels (∆*E*_10_). Afterward, ∆*E*_10_ in Figure 3(b) and (c) can be obtained as13 meV and 27 meV, which are of the same order of magnitude as in Figure 3(b) and (c). These results prove the appearance of strain-induced Landau levels. According to these simulation results, even if the pseudo-magnetic field is nonuniform, photonic Landau levels still exist in the band diagram. The appearance of photonic Landau levels and the size of the local bandgap is strongly related to the magnitude of the pseudo-magnetic field. Such dependences satisfy the properties of the quantum Hall effects. On top of that, the Lorentzian-distributed function gives a large flexibility to tune the peak position and maximum strength of the pseudo-magnetic field and exhibits tunable capability in photonic gaps by strain-engineering.

### 3.2 Topological edge state in the chiral structure

**Figure 4:**
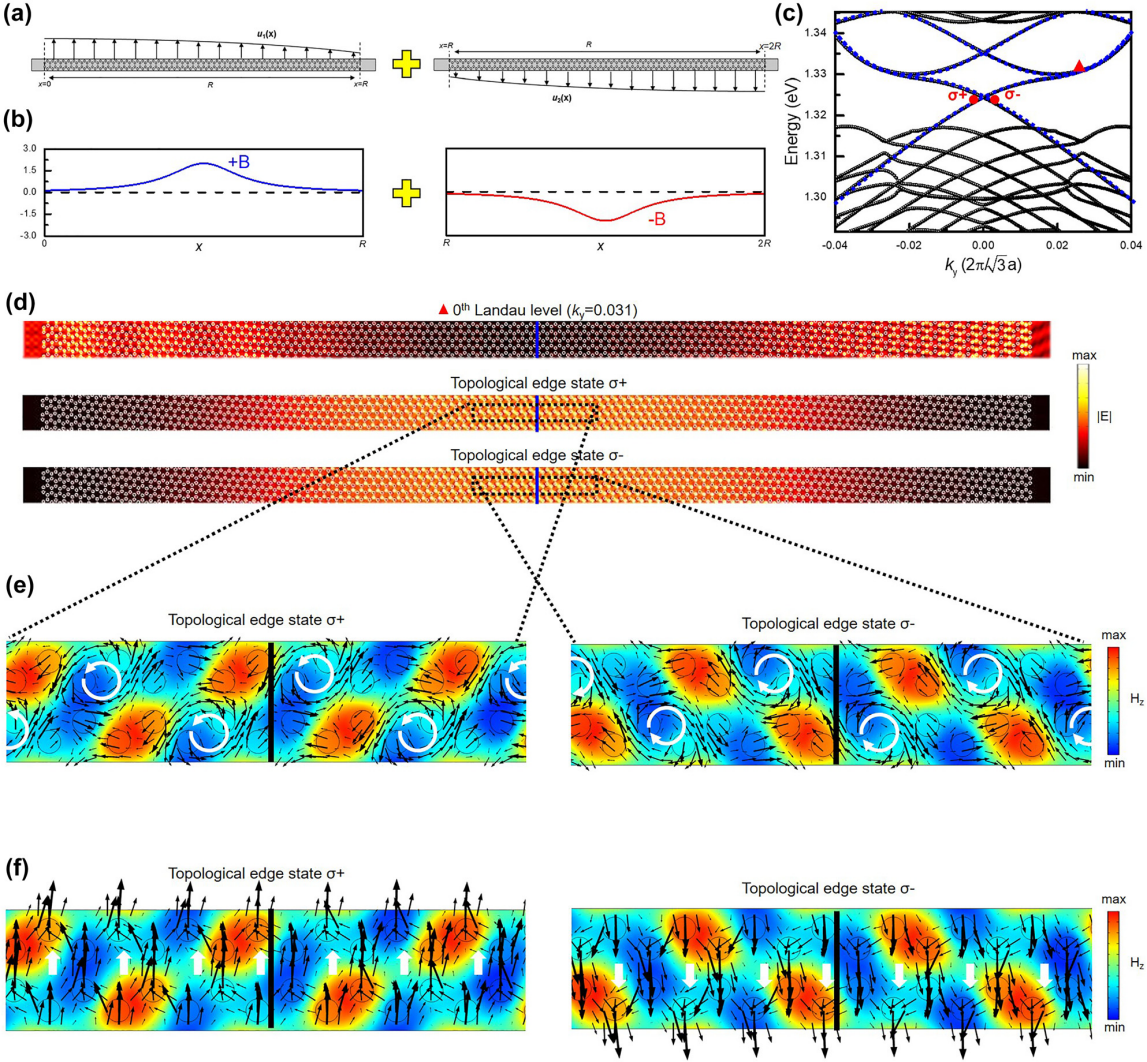
Strain-induced topological edge states. (a) The schematic diagram of the combination of the chiral structure, which is composed of two strained patterns with (b) the pseudo-magnetic field in opposite directions. (c) The band diagram of the chiral structure, which fixes *u*_max_ as 500 nm. (d) The corresponding electric field distribution of topological edge state *σ*+ and *σ−* signed as circular points in c, and the electric field distribution of the 0th photonic Landau level at 0.031 of *k*_*y*_ signed as a triangular point in (c). (e) The distribution of *H*_*z*_ component, where the black arrows indicate the distribution of the in-plane electric field. (f) The distribution of *H*_z_ component, where the black arrows indicate the distribution of the Poynting vector.

After demonstrating photonic Landau levels, we can go further to realize topological edge states. An idea to produce the topological edge state with an ultrastrong mode localization is to introduce an inflection point into the displacement function. Therefore, the displacement in Figure 2(c) is duplicated and then rotated 180° related to the coordinate: (*x* = *R*, *y* = $\sqrt{3}a/2$ ) to constitute the displacement distribution with an inflection point, whose schematic diagram of the combination and the corresponding second derivative are shown in Figure 4(a) and (b), respectively. In the light of the derivation in Eq. (4), the two combining structures separately produce the positive and negative pseudo-magnetic field, which leads to the reverse strain-induced quantum Hall effect and opposite topologies in photonic bands. Consequently, through this structural combination, a chiral pattern is constructed, and the combining boundary located at *x* = *R* is regarded as the demarcation line of two reverse quantum Hall effects. Figure 4(c) shows the band diagram of the chiral structure. It is astonishing to see that two crossing bands appear between the 0th and *C* = ±1 photonic Landau levels. Based on the discussion in Figure 1, it can be intuitive to understand that these crossing bands are constituted by topological edge states at K and K′ valley. As *k*_*y*_ is gradually shifted from the Γ point, photonic Landau levels emerge again owing to its state conversion, and the corresponding electric fields are distributed in the large strain region, as shown in the lowest figure of Figure 4(d). For the lower crossing band, the corresponding electric field distributions of topological edge state *σ*+ and *σ*− indicated at point *σ*+ and *σ*− in Figure 4(c) are shown in Figure 4(d). Notably, these states are localized at the combining boundary, which is beneficial for reducing the diffraction loss. Most crucially, to further discuss the topological characteristics, the correlated in-plane electric field vector and Poynting vector are also demonstrated in the distribution of black arrows in Figure 4(e) and (f), respectively. The governing equation we solved in the simulation model is simplified in the transverse electric (TE) form, so the dominated component of the magnetic field is in the *z*-direction *H*_*z*_, and the *H*_*z*_ distributions of topological edge state *σ*+ and *σ*− in the black-dash box both marked in Figure 4(d) are also shown in Figure 4(e) and (f). Apparently, by observing the corresponding polarization and power flow, the photonic spin-up state can be analogized to topological edge state *σ*+ owing to the left-hand circular polarization (LCP), and its propagation direction is in the +*y*-direction. On the contrary, topological edge state *σ*− is the right-hand circular polarization (RCP), which can also analogize the photonic spin-down state, and the propagation direction is in the −*y*-direction. Note that in photonic systems, the fields can point in the propagating directions because of nonuniform dielectric functions. Here the transverse circular polarizations are defined for a wave (propagating in the *y*-direction) with the fields rotating in the *x*–*y* plane. These results have exhibited robust evidence for the strain-induced topological edge state in spin-momentum locking. Furthermore, the angular momentum ***J*** has been introduced to calculate the effect of the strain-induced pseudo-magnetic field, whose equation is demonstrated in the following [39]:

(7) $$\vec{J}=\int \frac{\vec{r}\times \left(\vec{E}\times \vec{B}\right)}{4\pi c}dA$$

where ***r*** and ***c*** represent the spatial coordinate and speed of light, respectively. By introducing Eq. (7), the angular momentum of topological edge state *σ*+ and *σ*− are separately calculated as a positive and negative value in the *z*-direction, whose sign is determined by the LCP or RCP of the topological edge state. The detailed calculated values of ***J*** are also discussed in the Supplementary Material. Most vitally, pivotal evidence to prove that the edge state can be only created by the specific structural combination. Figure s1a shows the displacement distribution after the structural combination shown in Figure 4(a) and (b), which is composed of the positive and negative pseudo-magnetic field but setting *L* as R in Eq. (5). Besides, a displacement distribution composed of two positive pseudo-magnetic fields is constructed and shown in Figure s1b. The corresponding band diagrams of the structures arranged by the displacement function in Figure s1a and b are shown in Figure s2a and b, respectively. The constitution of the positive and negative pseudo-magnetic field can support the topological edge state, which proves that the strain-engineering we propose is an effective way to produce the opposite pseudo-magnetic fields and reverse the band topologies. Moreover, according to the topological natures, topological protection makes these topological edge states robust against smoothly varying defects or experimental imperfection, which do not create intervalley couplings. To examine the topological protection, we simulated the systems with twistedly perturbated structures and 60-degree-turn structures. In addition, the simulations show that these topological edge states can propagate in the zigzag direction. These results confirm the robustness of the strain-induced topological edge states. More detailed discussions are shown in the Supplementary Material and Figures s5 and s8.

### 3.3 The tunable ability of the strain-induced pseudo-magnetic field

**Figure 5:**
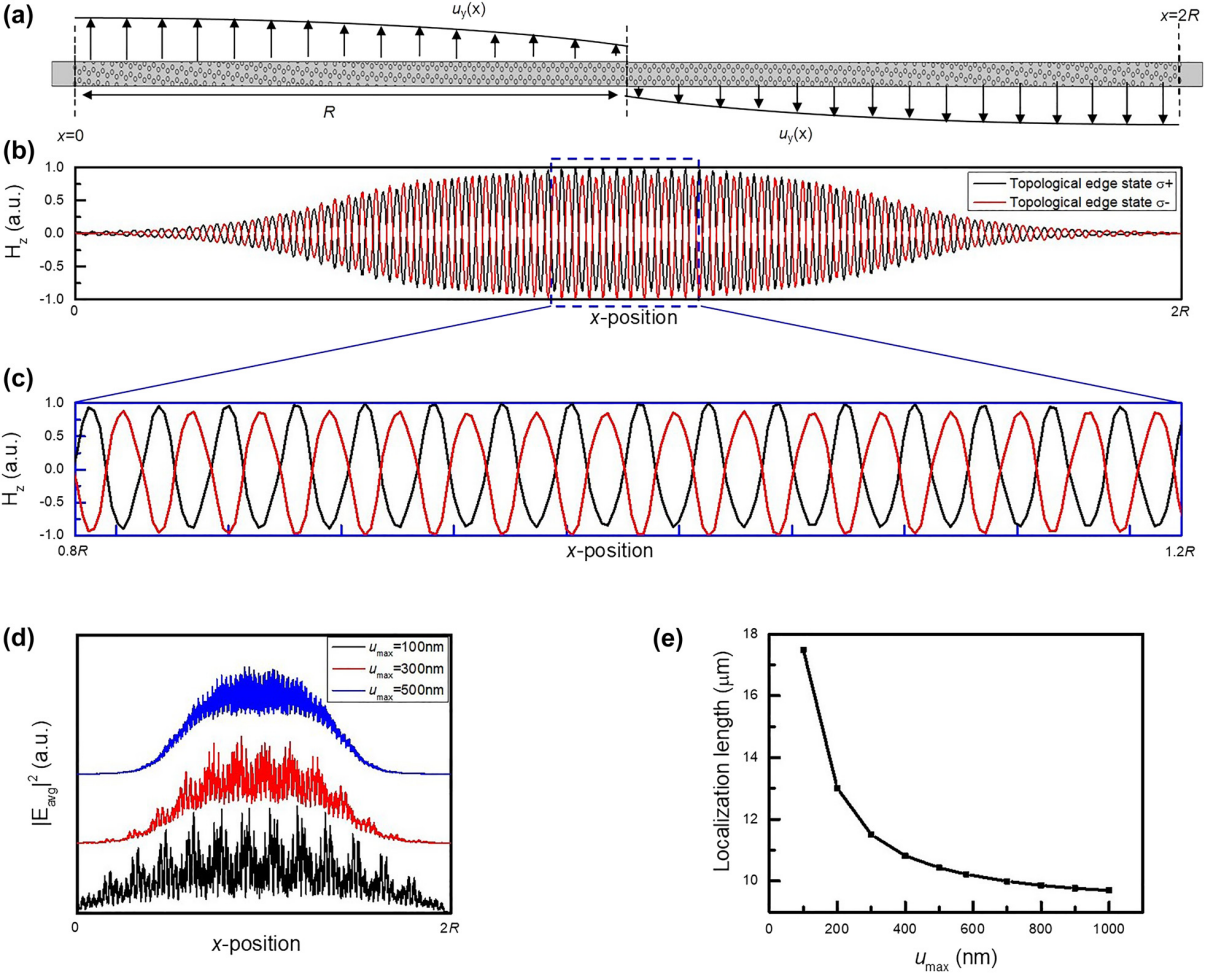
The parity of topological edge states and the tunability based on the strength of the pseudo-magnetic field. (a) The supercell of the chiral structure, which has Ander periods in the *x*-direction and maintains periodic in the *y*-direction. (b), (c) The distribution of *H*_*z*_ component in the *x*-direction, which is averaged along the *y*-direction in a supercell. The black and red line represent the edge state *σ*+ and *σ−* shown in Figure 3, respectively, and the enlarged blue dash box is shown in c. (d) The |*E*_avg_|^2^ of edge state *σ*+ in *u*_max_ = 100 nm, 300 nm, and 500 nm. (e) The mode localization as the function of *u*_max_.

According to the quantum Hall effect in electronics, the energy splitting between Landau levels is proportional to the external magnetic field. When a strong magnetic field is applied, the large energy gap provides an extraordinary insulating capability in the bulk region, causing the topological edge states to be highly localized at the structural boundary. As to the strain-engineering synthetic gauge field, if *u*_max_ in Figure 2(c) is increased, the local *u*_*y*,*xx*_ is increased too, resulting in a significant enhancement of the pseudo-magnetic field based on Eq. (4). Therefore, the strength of the pseudo-magnetic field is mainly dependent on *u*_max_. To verify the strain-induced quantum Hall effect, *u*_max_ in the chiral structure shown in Figure 5(a) is tuned to observe the variation of the localization length in the topological edge state. The equation of the localization length derived from the Anderson localization is shown in the following [40], [41], [42]:

(8) $$\text{Localization length}={\left[\frac{\int {\left|E_{\text{avg,lowpass}}\right|}^{4}dx}{(\int {\left|E_{\text{avg,lowpass}}\right|}^{2}d{x)}^{2}}\right]}^{-1}$$

where *E*_avg,lowpass_ is the electric field averaged along the *y*-direction in a supercell after passing a low-pass filter, and the detailed calculation is discussed in Figure s4. In addition, the chirality is discussed to explore the relation between the two topological edge states. Figure 5(b) shows *H*_*z*_ of topological edge state *σ*+ and *σ*− as the function of the *x*-coordinate, which is also calculated by averaging along the *y*-direction in a supercell, and the enlarged blue dash box is also shown in Figure 5(c). It is clear to see that topological edge state *σ*+ and *σ*− have the opposite chirality, which implies these states are correlated with each other. Afterward, the *u*_max_-dependent |*E*_avg_|^2^ distribution of topological edge state *σ*+ is demonstrated to discover the strength effect of the pseudo-magnetic field in Figure 5(d). As *u*_max_ is increased, the topological edge state is gradually confined to the combining boundary (*x* = *R*). To further quantify the increased confinement, the localization length is also calculated by Eq. (8) and shown in Figure 5(e). Remarkably, the increment of *u*_max_ can dramatically enhance the mode localization, and the localization length is varied from 17.5 µm to 9.69 µm as increasing *u*_max_ from 100 nm to 1000 nm. The strong localized feature makes the continuous strain modulation exhibit the great potential to reduce the diffraction loss near the structural boundary and improve the stability of topological edge states.

**Figure 6:**
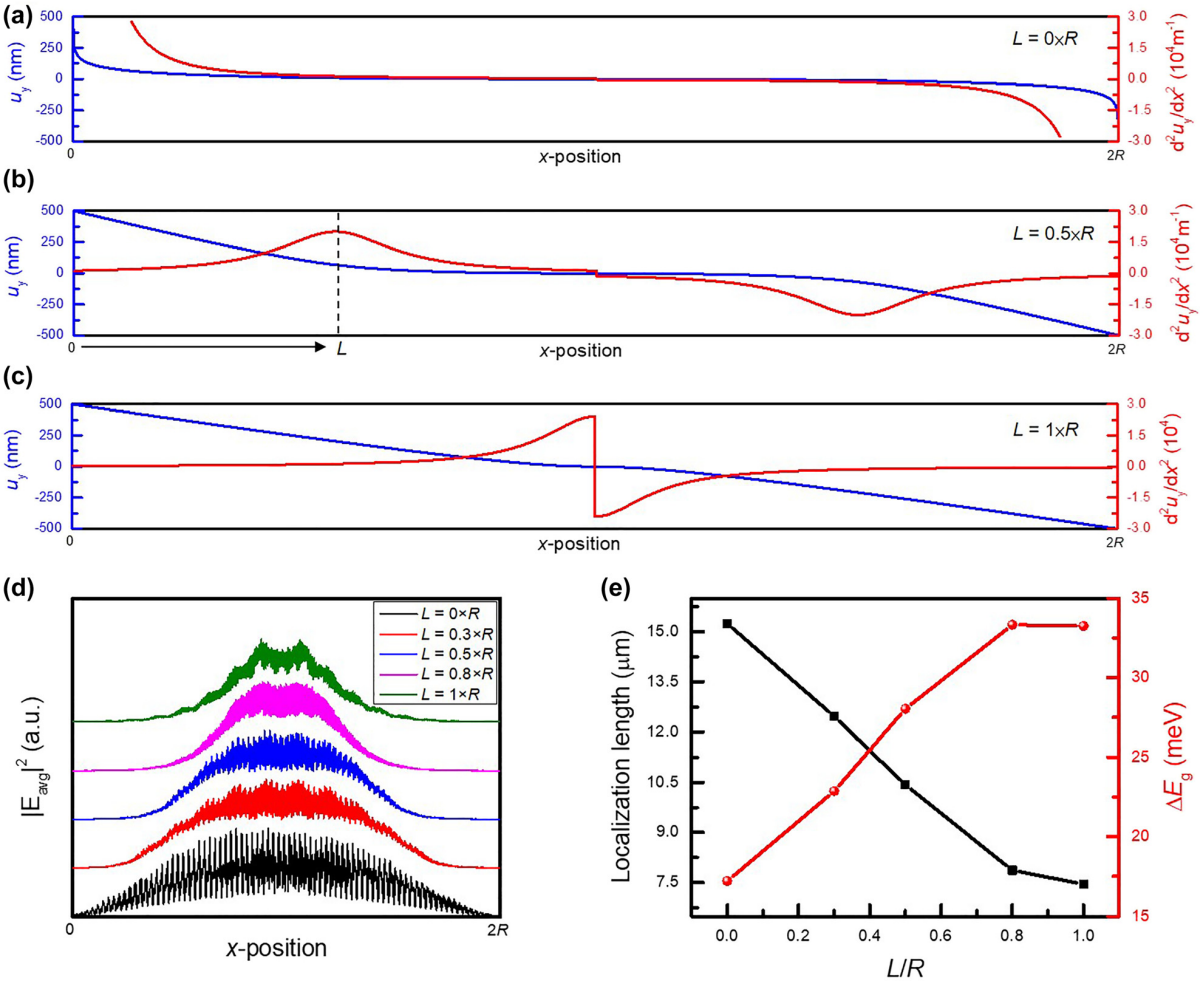
The tunability based on the peak position of the pseudo-magnetic field. (a)–(c) The continuous displacement function in different peak position of the pseudo-magnetic field, including *L* = 0 (a), 0.5 × *R* (b), and 1 × *R* (c). (d) The |*E*_avg_|^2^ of topological edge state *σ*+ in *L* = 0 × *R*, 0.3 × *R*, 0.5 × *R*, 0.8 × *R*, and 1 × *R*. (e) The mode localization and ∆*E*_g_ as the function of *LR*.

Furthermore, the mode localization is sensitive to the peak position of the pseudo-magnetic field. Figure 6(a)–(c) shows the displacement distribution as setting *L* as 0, *R*/2, and *R* in Eq. (5). If the peak position of the local responded pseudo-magnetic field is gradually away from the combining boundary, the mode profile of the topological edge state will be extended owing to the awful insulating capability in the bulk region. Consequently, as *L* is close to the combining boundary as shown in the *L*-dependent |*E*_avg_|^2^ distribution in Figure 6(d), the mode is rapidly converged to the center position, and the localization length in Figure 6(e) is shrunk to 7.45 µm when *L* becomes *R*. More interestingly, ∆*E*_g_ is also affected by the peak position of the pseudo-magnetic field, as shown in the red line of Figure 6(e), which shows that the strain-induced quantum Hall effect becomes more significant as the pseudo-magnetic field approaches the boundary. The design method proposed here provides an unprecedented way to architect a tunable topological edge state with the capability to achieve an ultrastrong localization, which also exhibits the great potential to be realized in practical application.

## 4 Conclusion

We studied topological photonic structures constituted by a chirally strained honeycomb lattice. We show that strain induces tunable gauge fields, which create Landau levels. At the boundary of two domains with opposite pseudo magnetic fields, robust topological edge states appear at the boundary. The edge states correspond to crossings in the original band gaps. On the contrary, no edge states appear at the boundary of two domains with pseudo magnetic fields of the same sign. The edge states have topological origin and are spin-momentum locked. Compared with other strain-induced topological photonic designs, smooth chiral strain-engineering gives rise to localized edge states with tunable capability. We showed that the confinement of the edge states is enhanced by modifying local strain distribution. We also quantify the corresponding increment of the degree of mode localization. Surprisingly, the localization length of the edge states can be reduced below 7.45 µm when choosing the *L*/*R* as 1 and *u*_max_ above 500 nm in Eq. (8). Hence, strongly localized topological edge states can be observed when the peak position of an intense pseudo-magnetic field is at the boundary of two domains. In photonic systems, strain can be designed during fabrication. Therefore, controlling the lattice displacement affects the strength of modulation and produces a pseudo magnetic field. This approach applies to photonic crystals regardless of their frequencies and sizes and hence enables broadband applications.

The proposed chiral strain-engineering topological photonics has excellent potential in advanced sensing and other applications. One can imagine innovative information processing devices such as filters and resonators in lasers and quantum optical systems.

## Supplementary Material

Supplementary Material
