# Supplementary material for: Pattern-tunable synthetic gauge fields in topological photonic graphene
Source: Nanophotonics. 2022 Mar 10;11(7):1297–308. doi: 10.1515/nanoph-2021-0647 (PMC11501643; doi:10.1515/nanoph-2021-0647)
Supplement: Supplementary file 1 — Supplementary Material [file j_nanoph-2021-0647_suppl_001.docx]

Supplementary Material

Zhen-Ting Huang^1^, Kuo-Bin Hong^1^, Ray-Kuang Lee^2,3^, Laura Pilozzi^4,5^, Claudio Conti^5,6^, Jhih-Sheng Wu^*,1^ and Tien-Chang Lu^*,1^

**Pattern-tunable synthetic gauge fields in topological photonic graphene**

^1^Department of Photonics and Institute of Electro-Optical Engineering, College of Electrical and Computer Engineering, National Yang Ming Chiao Tung University, Hsinchu 30050, Taiwan

^2^ Institute of Photonics Technologies, National Tsing Hua University, Hsinchu 30013, Taiwan

^3^ Physics Division, National Center for Theoretical Sciences, Hsinchu 30013, Taiwan

^4^ Institute for Complex Systems, National Research Council (ISC-CNR), Via dei Taurini 19, 00185 Rome, Italy

^5^ Research Center Enrico Fermi, Via Panisperna 89a, 00184 Rome, Italy

^6^ Department of Physics, University Sapienza of Rome, Piazzale Aldo Moro 5, Rome 00185, Italy

*Correspondence and requests for materials should be addressed to:

[timtclu@nycu.edu.tw](mailto:timtclu@nycu.edu.tw) (T.C.L.) and [jwu@nycu.edu.tw](mailto:jwu@nycu.edu.tw) (J.S.W.)

**Keywords:** chiral strain-engineering, synthetic gauge fields, strong localization, topological edge state, tunable capability

- **The valley Chern number in each photonic Landau level**

As the strain-induced is applied, the effective Hamiltonian near a valley in the strained system can be given by equation (2) of the main text. The Berry connection (*A*_c_) and Berry curvature (Ω) can be further derived by equations (s2) and (s3) [1-3].

$A_{c}(k)=-i<\psi(k)|\nabla_{k}|\psi(k)>$, (s1)

$\Omega(k)=\nabla_{k}\times A_{c}(k)$ , (s2)

where $\psi(k)$ is the eigenstate of equation (2). The valley Chern number of this specific valley is obtained by

$C=\int\Omega(k)d^{2}k$ (s3)

Due to time-reversal symmetry, the sum of the valley Chern numbers over all valley vanishes.

Hence, the valley Chern numbers are defined for each valley, respectively.

- **The verification of topological edge states in the chiral structure**


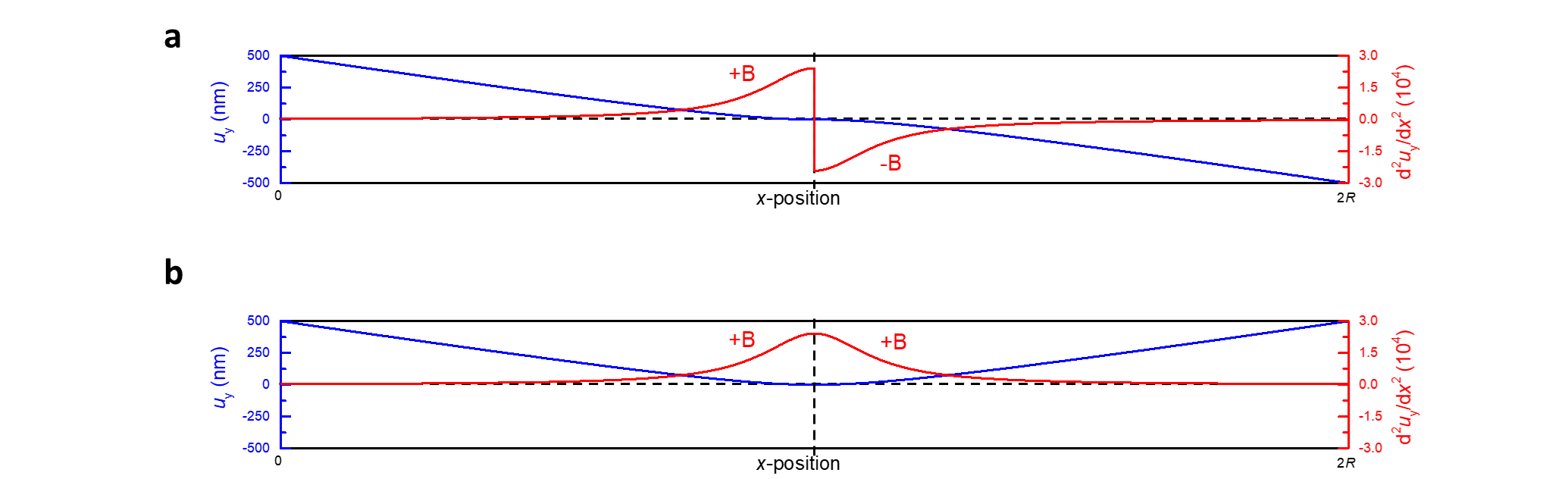


**Figure s1:** The verification of topological edge states. (a),(b) The continuous displacement function after the structural combination of positive/negative (a) or positive/positive (b) pseudo-magnetic field, where *u*_max_ and *L* are set as 500 nm and 1×*R*, respectively. The blue line indicates the distribution of *u*_y_, and the red line indicates the distribution of $\partial^{2}u_{y}/\partial x^{2}$.

To verify the formation mechanism of topological edge states induced by our proposed chiral structure, two displacement functions are considered and shown in Figure s1. First of all, Figure s1a shows the displacement distribution after a combination of two patterns with positive and negative pseudo-magnetic field, which is the same as Figure 6c in the main text. The opposite sign of the pseudo-magnetic field causes the reverse quantum Hall effect in the chiral structure, leading to a reverse topology in photonic Landau levels. Then, the topological edge state would exist between the two combining patterns. Figure s1b shows another kind of structural combination, which is composed of two patterns both with the positive pseudo-magnetic field. Owing to the same sign of pseudo-magnetic field, the topology of photonic Landau levels in two combining patterns is the same. Consequently, there should be no topological edge state located at the central boundary. By calculating band structures in these two structural combinations, the formation of our proposed strain-induced topological edge states can be verified. Furthermore, the photonic angular momentum introduced in equation (7) in the main text is also calculated to ensure the topological characteristics. If edge states in the chiral structure belong to the topological edge state, the spin-momentum locked condition must be satisfied. Namely, the angular momentum of edge states should be in the opposite direction, which is owing to the reverse spin and orbital propagation. In our calculation, the angular momentum of topological edge state σ+ and σ- in Figure 4 are separately calculated as 5.282×10^-12^ $\text{J}\cdot\text{s}$ and -5.129×10^-12^ $\text{J}\cdot\text{s}$ in the *z*-direction, which verifies the topological nature.


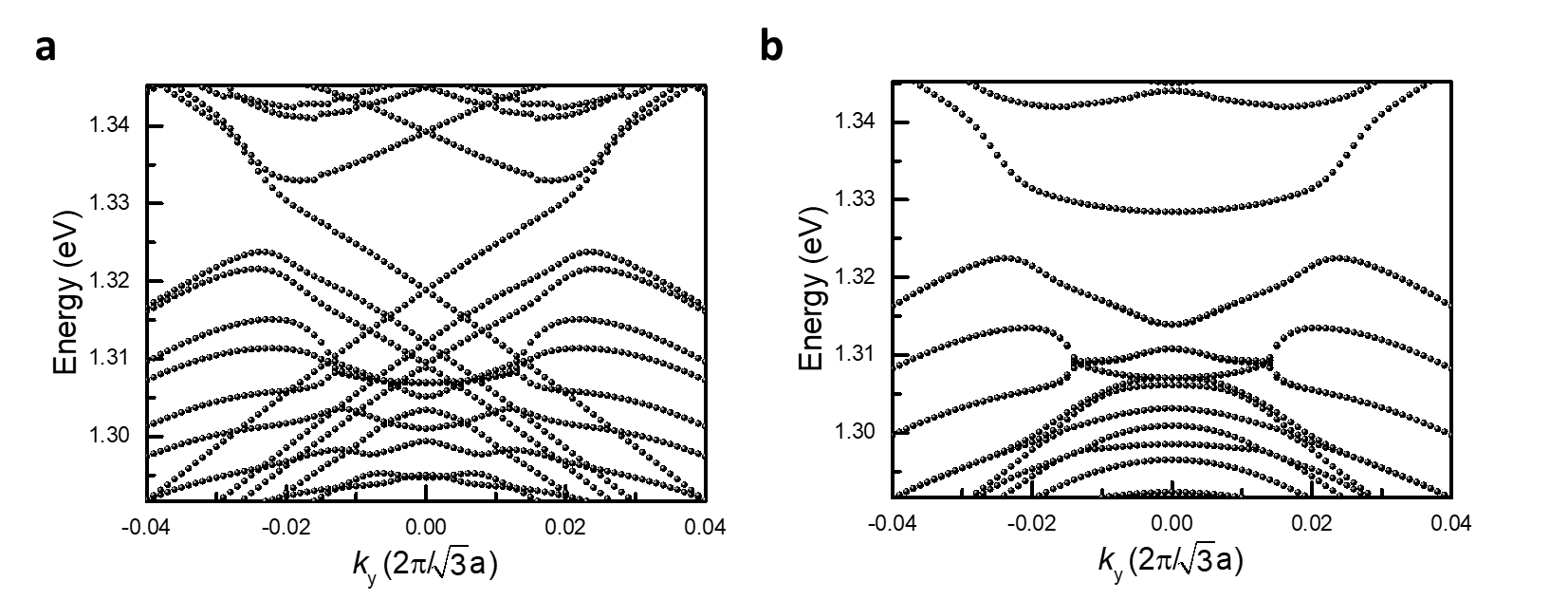


**Figure s2:** The verification of topological edge states. (a),(b) The band diagram of the chiral structure, which is obtained by combining two patterns giving positive/negative (a) or positive/positive (b) pseudo-magnetic field.

Figure s2a and b demonstrate the corresponding band diagram of the structure with the displacement shown in Figure s1a and b, respectively. Notably, in Figure s2a, there are two crossing bands inside the local bandgap, located between photonic Landau levels, which satisfy the characteristics of strain-induced topological edge states discussed in the main text. On the contrary, in Figure s2b, there is only a flat band inside the local bandgap, which belongs to a defect mode produced by the combination of two patterns. Although its spatial distribution is also located near the combining boundary, this mode cannot propagate along the *y*-direction, a feature inferable by its slope shown in the band diagram.

- **The filtration of supermode bands in the finite-element method**


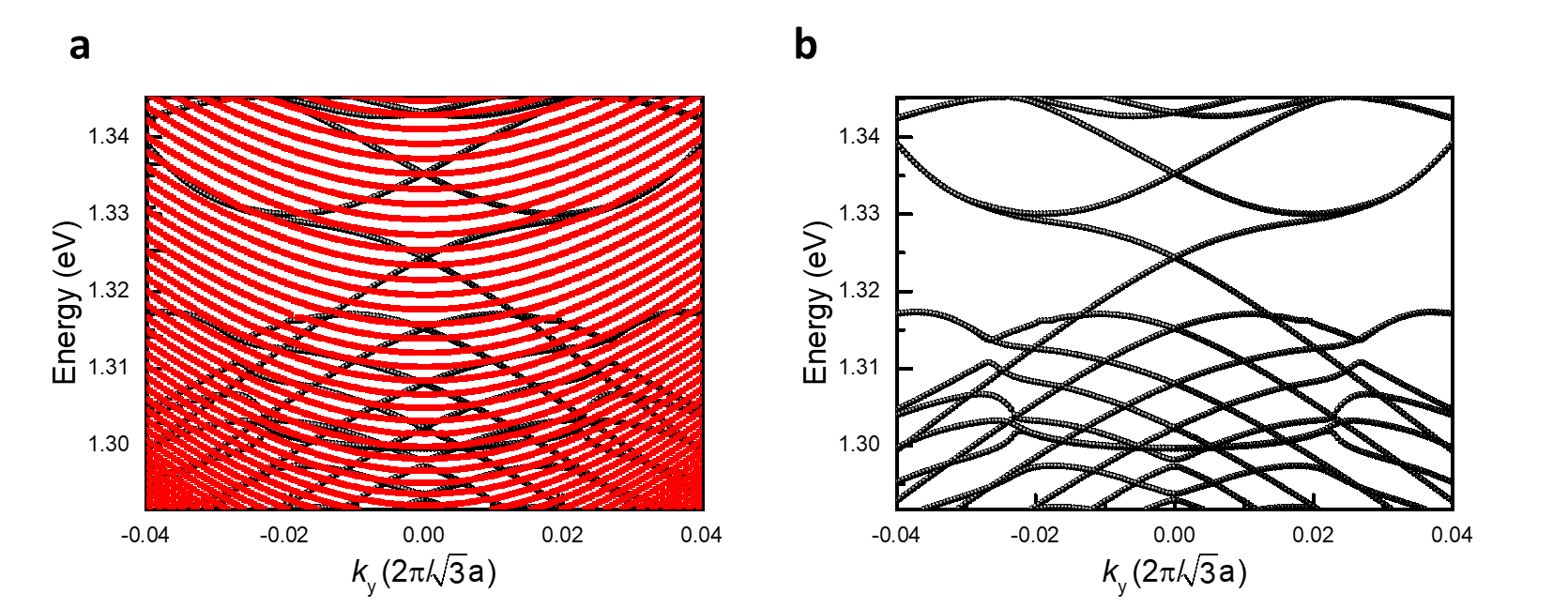


**Figure s3:** The filtration of supermode bands. (a),(b) The calculated band diagram before (a) and after (b) the filtration of supermode bands. The red bands indicate the calculated supermode bands.

In this paper, we use a finite-element software (COMSOL Multiphysics) to perform the simulation. As a result of the large supercell, supermodes of low quality factors appear, as shown in the red and dense curves in Figure s3a. These supermodes belong to the higher-order bulk modes, which have more complicated transverse profiles [4]. Typically, these modes have lower quality factors due to coupling to the outside of the lattice. Therefore, we can use a quality factor filter to remove these modes. To simplify our calculation, we use some methods introduced in Figure s3 to filtrate these unwanted bands. Those supermode bands appearing in the band structures can be separated from our simulation through a quality factor filter because these bands show relative low quality factors. Figure s3a is the calculated band diagram shown in Figure 4c in the main text. Before filtrating, the solutions show many bands with relative low quality factors inside the local bandgap, making it difficult to observe the band-crossing feature of topological edge states. After the filtration, the two striking crossing modes clearly appear inside the local bandgap, as shown in Figure s3b. Consequently, we remove these modes in the main text.

- **The calculation of localization length**

**Figure s4:** The calculation with a low pass filter. The |*E*_avg_|^2^ distribution of topological edge state σ+ when *u*_max_=500 nm and *L*=0.5×*R*.

To estimate the localization length introduced in equation (8) in the main text, a low-pass filter is used to obtain *E*_avg,lowpass_ by suppressing the fluctuation resulted from the sampling accuracy. Figure s4 is the |*E*_avg_|^2^ distribution of topological edge state σ+ mentioned in Figure 4c with and without a low-pass filter, which only allows passing the fundamental frequency. After the low-pass filter, we can obtain a clear distribution of *E*_avg,lowpass_.

- **The robustness of the stain-induced topological edge states**

**
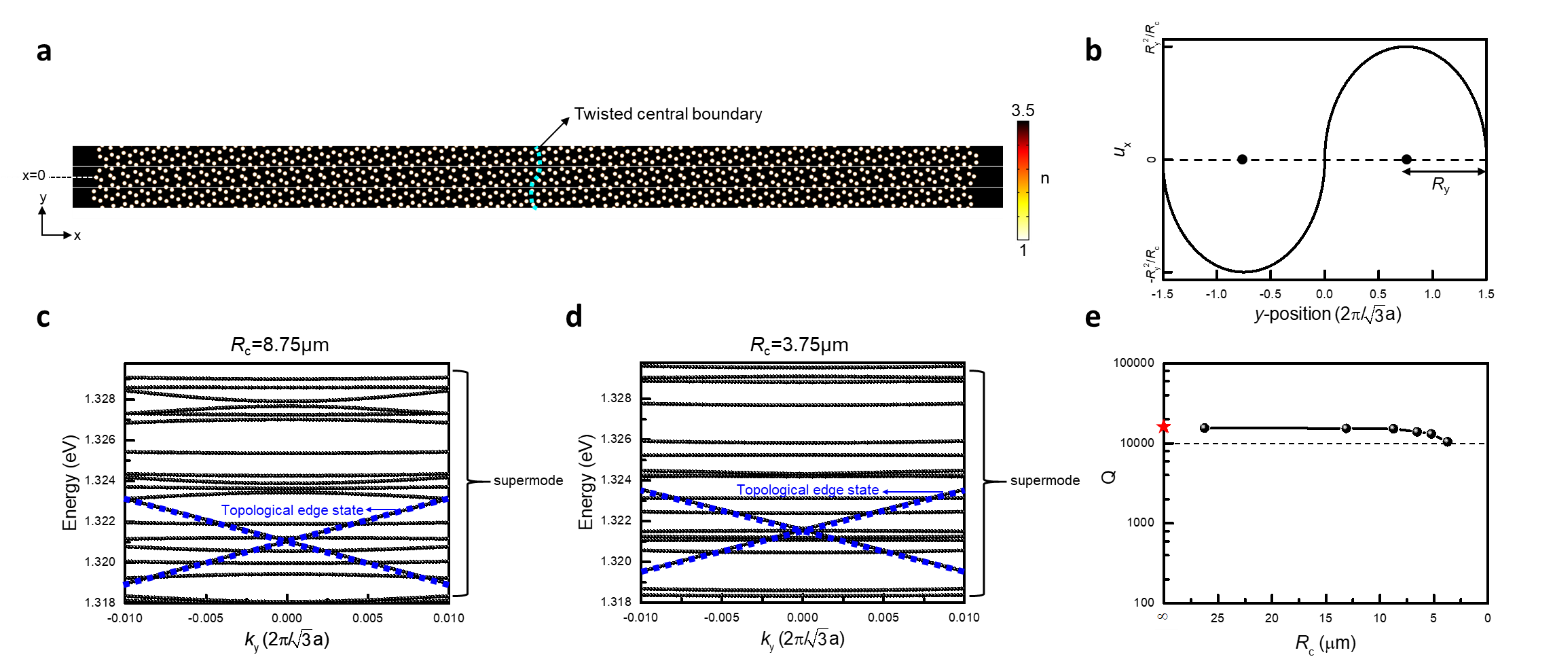
**

**Figure s5.** The simulation for testing the robustness of the strain-induced topological edge state. (a) The chiral structure with *u*_max_=500 nm and *L*=1×*R*. Most specially, a twisted central boundary followed by (b) a *y*-varied *x*-displacement function is added, where *R*_c_ represents the minimum radius of curvature in the twisted chiral structure. (c),(d) The band diagrams with *R*_c_ of 8.75 µm (c) and 3.75 µm (d) are demonstrated, where the blue dot lines indicate the bands of topological edge states. (e) The quality factor *Q* as the function of *R*_c_.

To test the scattering-immune performance of our strain-induced topological edge states, a *y*-varied *x*-displacement function is considered in the lattice arrangement, which is demonstrated in the following:

$u_{\text{x}} = \frac{R_{y}}{R_{c}}\times\text{Re}[\sqrt{R_{y}^{2}-(y-R_{\text{x}})^{2}}-\sqrt{R_{y}^{2}-(y+R_{\text{y}})^{2}}]$ (s4)

Where *R*_c_ represents the minimum radius of curvature in the twisted chiral structure. Figure s5a and b show the twisted chiral structure and equation (s4), respectively. According to the topological natures, topological edge states must be robust against the deformation. Even though the structure is twisted, topological edge states should still exist, and the quality factor should be also slowly varying and maintained at a certain value. In this case, a supercell with *R*_y_=0.75($2\pi a/\sqrt{3}$) is used in the simulation model. Remarkably, as the chiral structure is twisted more significantly, which corresponds to a lower *R*_c_ in equation (s4), the crossing of the topological edge states only shifts slightly, as shown in Figure s5c and d, and the band crossing still maintains. Figure s5e shows the quality factors of topological edge states as the function of *R*_c_. It is worth noting that the variation of quality factor is small, and all of that is above 10000 and close to the result of the untwisted chiral structure. This calculation proves the robustness of the strain-induced topological edge state and shows how stable these states are against the deformation.

- **The gauge field and calculation of the material-related parameter**

**
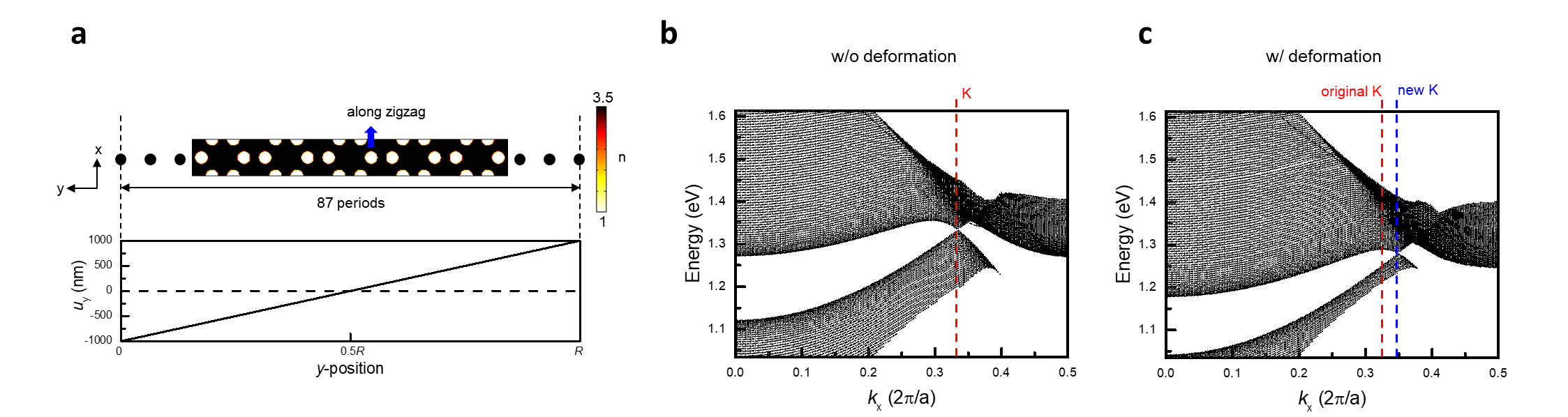
**

**Figure s6.** The effect of the strain-induced gauge field. (a) The refractive index profile of a honeycomb superlattice, which is finite along the *y*-direction (armchair) and periodic along the *x*-direction (zigzag), and a linearly distributed displacement function is applied in the arrangement of air holes. (b),(c) The band diagrams of the honeycomb superlattice without (b) and with (c) the deformation.

According to the effective Hamiltonian near one of the valley shown in equation (2), the strain-induced gauge field will produce a shift to the position of K valley. To verify that the pattern-tunable strain proposed in this paper is capable of inducing a synthetic gauge field, a linearly distributed displacement function, which is *u*_y_ function of y, is added in the arrangement of air holes in a honeycomb superlattice, as shown in Figure s6a. This displacement mimics the deformation of the photonic graphene along the armchair, and it would induce a constant gauge field in the *x*-direction (*A*_x_) based on equation (1) and (3). Figure s6b shows the band diagram before applying the displacement, a clear valley can be observed, and the group velocity at the Dirac point (*v*_D_) is calculated by its slope, which is 7.8×10^7^ m/s. When applying the displacement, the position of K valley is shift form 0.335 (2π/a) to 0.35 (2π/a), as shown in Figure s6c. Subsequently, based on equation (2), *A*_x_ is determined by this shift, which is 0.015 (2π/a). After dividing *A*_x_ by the slope of the displacement function, the material-related parameter (*g*_2_) can be determined to be 0.228 (2π/a).

- **The band diagram of the gapless edge state**

**
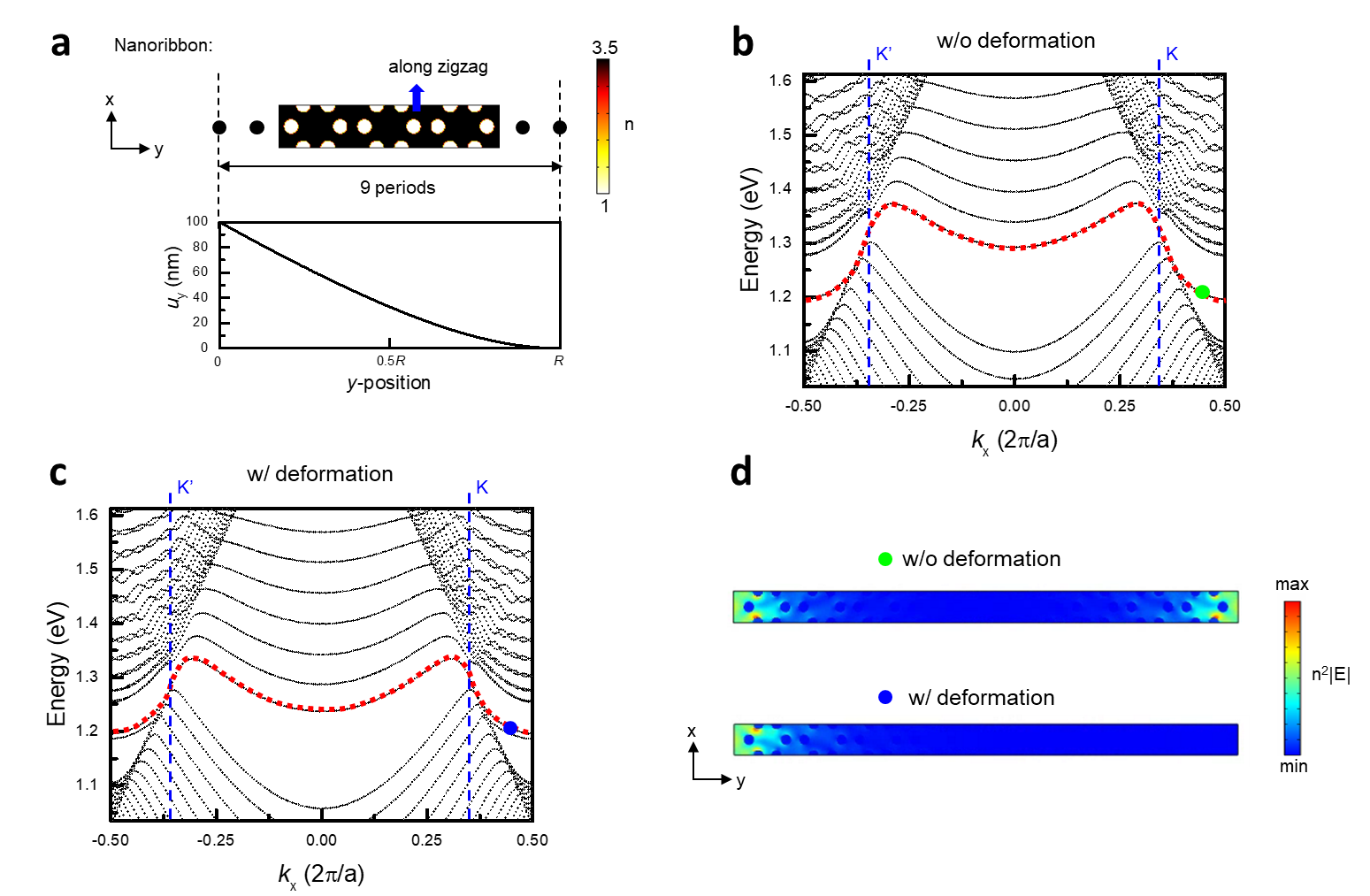
**

**Figure s7.** The band diagram of the gapless edge state in a short nanoribbon. (a) The refractive index profile, which is only 9 periods in the *y*-direction (armchair) and periodic along the *x*-direction (zigzag). The displacement function shown below follows the derivation introduced in Figure 2c but changes the variable in equation (5) to y and sets *L* as *R*. (b),(c) The band diagram of the short nanoribbon without (b) and with (c) the deformation, and the red dotted line indicates the dispersion of the gapless edge state. (d) The corresponding electric field distribution of the green and blue points indicated in b and c (*k*_x_=0.44 (2π/a)).

For a graphene nanoribbon, which is usually fabricated by cutting the complete graphene, and its transverse width is far shorter than the longitudinal length, gapless edge state always exists at the boundary no matter how short the nanoribbon is and propagates along zigzag [5]. Therefore, we use a honeycomb superlattice, which is only 9 periods in the *y*-direction and periodic along the *x*-direction, to mimic the photonic graphene nanoribbon, as shown in Figure s7a. Also, a displacement function, which follows the derivation discussed in Figure 2, is added in the arrangement of air holes to explore the influence of strain on the gapless edge state. Figure s7b and c show the band diagram of the short nanoribbon without and with the deformation. It is obvious to see that the gapless edge state is located inside the bandgap, and its dispersion is nearly not affected by the deformation. Figure s7d shows the electric field distribution of the gapless edge state, and the electric field of the lower inset becomes asymmetric between two ends of the nanoribbon because of the asymmetric deformation.

- **Turning the propagation direction of topological edge states**

**
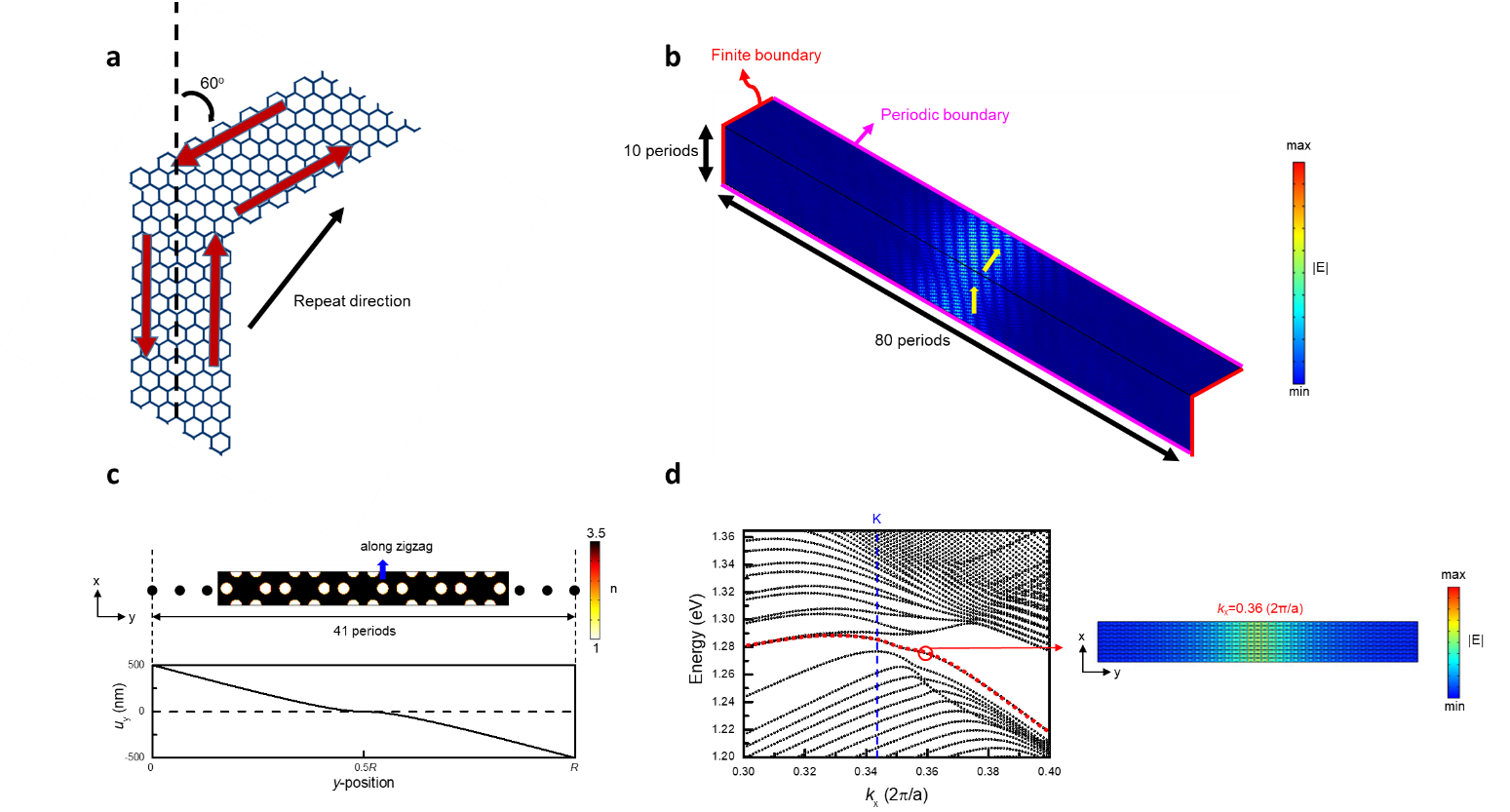
**

**Figure s8.** Turning the propagation direction of the strain-induced topological edge state. (a) Schematic diagram of the structure with the 60-degree turning angle in the simulation model, where the red arrow indicates the direction of the applying displacement, and the displacement added in the arrangement of air holes is the same as Figure 6c but changes the transverse period number to 80 periods. (b) The corresponding electric field distribution of the topological edge state, where the yellow arrow indicates the propagation direction of the topological edge state. (c) The refractive index profile of a honeycomb superlattice, which is finite along the *y*-direction (armchair) and periodic along the *x*-direction (zigzag), and the displacement follows the derivation introduced in Figure 6c but changes the variable in equation (5) to y. (d) The band diagram near the K valley, where the red dotted line indicates the strain-induced topological edge state, and the right inset shows the electric field distribution.

To discuss more propagation properties of the strain-induced topological edge state, we build a simulation model to solve the state with a 60-degree turning angle, as shown in Figure s8a. Figure s8b shows the electric field distribution of the topological edge state, which belongs to the lower crossing band inside the local bandgap. It is obvious to see that the propagation direction is bent successfully. Furthermore, the strain-induced topological edge state can also propagate in the zigzag direction. Figure s8c shows a honeycomb superlattice, and the applied displacement will produce two pseudo-magnetic fields with different directions at two sides of the central boundary. Figure s8d shows the band diagram near the K valley. Remarkably, the strain-induced topological edge state appears inside the local bandgap, and its electric field is highly localized at the central boundary, which implies that by the strain we introduced in this paper, a robust and flexible topological edge state can be constructed, and it is beneficial in practical application.

**References**

[1] F. Zhang, A. H. MacDonald, and E. J. Mele, “Valley Chern numbers and boundary modes in gapped bilayer graphene.” *Proc. Natl. Acad. Sci. U.S.A.*, vol. 110, pp. 10546-10551, 2013.

[2] Z. Wang, Y. D. Chong, J. D. Joannopoulos, and M. Soljačić, “Reflection-free one-way edge modes in a gyromagnetic photoniccrystal.” *Phys. Rev. Lett.*, vol. 100, pp. 013905, 2008.

[3] D. Xiao, M. C. Chang, and Q. Niu, “Berry phase effects on electronic properties,” *Rev. Mod. Phys.*, vol. 82, pp. 1959-2007, 2010.

[4] M. Skorobogatiy, K. Saitoh, and M. Koshiba, “Transverse lightwave circuits in microstructured optical fibers: resonator arrays,” *Opt. Express*, vol. 14, pp. 1439-1450, 2006.

[5] A. Maffucci and G. Miano, “Electrical Properties of Graphene for Interconnect Applications,” *Appl. Sci.*, vol. 4, pp. 305-317, 2014.
